# Differential effects of mindfulness treatment and mobile neurofeedback on event-related potentials in early posterior negativity in cancer patients: a clinical-experimental parallel group design

**DOI:** 10.3389/fpsyg.2024.1395032

**Published:** 2024-10-01

**Authors:** Madeleine Fink, Kira Schmidt, Axel Kowalski, Saskia Pasche, Calvin Albrot, Marvin Krawutschke, Theresa Schweig, Mitra Tewes, Eva-Maria Skoda, Martin Teufel, Bernhard W. Müller

**Affiliations:** ^1^Clinic for Psychosomatic Medicine and Psychotherapy, LVR-University Hospital Essen, University of Duisburg-Essen, Essen, Germany; ^2^Center for Translational Neuro- and Behavioral Sciences (C-TNBS), University of Duisburg-Essen, Essen, Germany; ^3^West German Cancer Center (WTZ), LVR-University Hospital Essen, University of Duisburg-Essen, Essen, Germany; ^4^Department of Psychiatry and Psychotherapy, LWL Clinic Dortmund, Ruhr University Bochum, Dortmund, Germany; ^5^NeuroFit GmbH, Kempen, Germany; ^6^Mental Health Research and Treatment Center, Ruhr University Bochum, Bochum, Germany; ^7^West German Cancer Center (WTZ), Department of Medical Oncology, University Hospital Essen, University of Duisburg-Essen, Essen, Germany; ^8^Department of Palliative Medicine, University Hospital Essen, University of Duisburg-Essen, Essen, Germany; ^9^LVR-University Hospital Essen, Department of Psychiatry and Psychotherapy, Medical Faculty, University of Duisburg-Essen, Essen, Germany; ^10^Department of Psychology, University of Wuppertal, Wuppertal, Germany

**Keywords:** cancer, EPN, neurofeedback, EEG-biofeedback, mindfulness therapy, emotion regulation

## Abstract

**Introduction:**

Cancer frequently leads to psychological challenges, among them emotion regulation problems. These can be alleviated with the help of mindfulness therapies or neurofeedback (NF) interventions. Possible intervention effects on emotion procession can be detected in clinical EEG studies by exploring event-related potentials, e.g., early posterior negativity (EPN), which recently has been established to investigate emotional processing and represents very early attention to affective stimuli. Therefore, this clinical-experimental study investigated the efficacy of mindfulness and NF (10 sessions each) on the EPN in oncology patients.

**Method:**

The study enrolled 42 cancer patients (age: 31–73 years; gender: 28 female, 14 male). The study design was an RCT with a parallel group [NF (*n* = 21) versus mindfulness (*n* = 21)] waitlist paradigm. EEG recordings in an oddball task with neutral, rare positive and negative valence and high and low arousal stimuli were performed at three measurement time points (T0 = before waitlist, T1 = before intervention, T2 = after intervention). Following preprocessing, data from electrodes O1, Oz and O2 were analyzed for EPN amplitudes.

**Results:**

Response time did not differ across groups and conditions. Comparing EPN at T1 and T2, there was a significant interaction of time, valence, and intervention (*p* = 0.042). Descriptive statistics showed increased EPN for negative stimuli after the NF intervention (T1 to T2), while EPN for positive stimuli only slightly increased. For mindfulness, positive stimuli evoked stronger amplitudes after the intervention, while EPN for negative stimuli increased from T1 to T2.

**Conclusion:**

Distinct effects were observed for the EPN for pictures with negative valence. Here, it is presumed that mindfulness treatment led to a refocusing of attention with a focus on positive valence, whereas NF seems to entail a different processing of images with negative valence and is therefore to be seen more in the sense of a confrontational approach. Our results suggest that both interventions are suitable for modulating EPN. However, it is not clear to what extent the effects are due to the interventions alone and how other factors might have affected the amplitudes, which highlights the need for further research in this area.

## 1 Introduction

Though its mortality rate continues to decline, e.g., in Germany (Barnes et al., 2016), cancer is still the second leading cause of death worldwide with approximately 9.9 million deaths in 2020 (Sung et al., 2021). Currently, the number of cancer patients is increasing persistently and has nearly doubled in Germany since the 70s (Barnes et al., 2016). Due to better treatment options and the aging of the population, there is also a growing number of cancer survivors (De Moor et al., 2013). The United States report approximately 13.7 million cancer survivors, with a survival rate of 64% 5 years or longer after diagnosis, 40% 10 years or longer, and 15% 20 years or longer. It is concerning that many cancer patients and survivors suffer from severe psychological and physical clinical symptoms, such as severe distress and depressive symptoms, cognitive impairment, pain, and fatigue, caused by the disease and its treatment (Fink et al., 2023). In many cases, the psychological burden persists beyond treatment and manifests as long-term and late effects of cancer, which highlights the need for psycho-oncological follow-up care (Deimling et al., 2006).

### 1.1 Emotion regulation

Emotions refer to a hypothetical and complex construct that is considered to play an important role in intrapsychic experience and might also affect motivational processes and need satisfaction. Emotions fulfill a variety of functions, e.g., preparing behavioral responses, helping in the decision-making process of personally relevant events/situations, facilitating interpersonal interaction, and supporting memory in the recall of important events (Suri et al., 2023). However, emotions can also be dysfunctional and have a negative impact on health (Parrott, 1993). A growing body of research suggests that emotional dysregulation is heavily implicated in the etiology and maintenance of affective disorders (Cisler et al., 2010; Hofmann et al., 2012; Joormann and Quinn, 2014; Kashdan et al., 2008; Mennin, 2004; Vanderlind et al., 2020). Although approximately 35% of cancer patients experience psychological distress, very few studies investigated emotion dysregulation within this population (Zabora et al., 2001). Patients with affective symptoms often fulfil the criteria for a major depression (Reuter et al., 2004). However, since the symptoms of comorbid depressive disorders often overlap with somatic symptoms in cancer patients, it remains a challenge to accurately diagnosing comorbid depressive disorders in cancer patients. Affective symptoms in cancer patients include a depressive mood in form of sadness, loss of interest, hopelessness, lack of drive and feeling of guilt, as well as anxiety symptoms such as fear of the future or existential fears (Husson, 2013). Further, there is a notable lack of research on the specific patterns of depressive symptoms in cancer patients, which is essential for developing appropriate diagnostic protocols (Reuter et al., 2004). The concept of emotion regulation encompasses the strategies and techniques individuals employ to manage their emotions in response to stressors and depressive moods (Austenfeld and Stanton, 2004). Regarding these considerations and the similarity of the affective symptoms of cancer patients, further investigation of emotional regulation deficits would contribute to a better understanding of the condition but also consequently of the treatment.

### 1.2 Early posterior negativity

The early posterior negativity (EPN), an event-related potential, has recently been established as a tool to investigate emotion processing and represents early attention to emotional stimuli (Gößwein, 2014). After the presentation of a stimulus, changes in the EPN amplitude usually occur approximately 150–300 ms later (Schupp et al., 2006). These changes include relatively greater negativity over occipital-temporal poles in the EEG for emotional stimuli compared to neutral stimuli (Gößwein, 2014). Brain regions responsible for primary and secondary visual processing are thought to be the source of the EPN (Olofsson et al., 2008). Further theoretical interpretations of the EPN imply that it is a natural selective attention - the evaluation of different image contents is guided by perception, and it is responsible for processing arousing and emotional stimuli (Olofsson et al., 2008). Other studies have found an almost reflexive processing of affective stimuli. This is reflected by the fact that the amplitude of the EPN changes after a very short presentation (200 ms) of an image with emotional content (Junghöfer et al., 2001). Accordingly, the EPN represents an important instance in the cognitive processing of stimuli. In a study by Schupp et al. (2007), subjects were instructed to direct selective attention to images that represented a specific valence (Schupp et al., 2007). This resulted in a stronger amplitude (negation) for both negative and positive images during the processing of the stimuli. These findings suggest that the EPN is particularly sensitive to arousing stimuli. Other studies came to similar conclusions: Stronger amplitudes were found for affective stimuli compared to neutral ones, but no dependence on the level of arousal was observed (Leite et al., 2012). However, valence and arousal usually have no effect on behavioral reaction times, which typically vary between 497 and 509 ms for neutral, positive, negative as well as high and low arousal stimuli (Conroy and Polich, 2007; Rozenkrants and Polich, 2008). However, Lu et al. (2017) observed an interaction between valence and arousal on reaction time, showing significantly shorter reaction times for low arousal positive images (*M* = 441.98, *SD* = 48.93) than for low arousal negative images (*M* = 455.74, *SD* = 50.57, *p* = 0.01) (Lu et al., 2017). To our knowledge, the application of EPN in cancer patients has not yet been investigated.

### 1.3 Mindfulness based treatments in cancer patients

The concept of mindfulness has its origins in the Buddhist religion, with the aim to overcome suffering and craving (Lohmann and Annies, 2018). In psychological and medical practice, mindfulness interventions, e.g., Kabat-Zinn’s Mindfulness-Based Stress Reduction (MBSR), were originally used as a treatment for chronic pain (Kabat-Zinn, 1982). Since it might alleviate other symptoms as well, MBSR has been used in the treatment of a number of conditions and adapted to prevent relapse in depression (Kuyken et al., 2008). In addition to that, there are positive effects of MBSR on emotion regulation and affective disorders, e.g., anxiety disorder and depression (Goldin and Gross, 2010). Mindfulness has already been found to reduce affective symptoms, such as rumination, in cancer patients (Boyle et al., 2017; Cillessen et al., 2018). Many of those symptoms can be found in cancer patients, which is why mindfulness is already widely used as a potential treatment modality in psycho-oncology with positive effects on various physical and psychological symptoms (Cillessen et al., 2019; Ledesma and Kumano, 2009; Victorson et al., 2020). Even though this method is very widely used, low acceptance and side effects are occasionally reported (Schlosser et al., 2019). The systematic review by Lomas et al. (2015) also showed that mindfulness was most commonly associated with increases in alpha and theta banding (Lomas et al., 2015), which is similar to the effects of neurofeedback (NF) interventions.

### 1.4 Electroencephalographic neurofeedback in cancer patients

Electroencephalographic neurofeedback (EEG NF) is a scientifically based, innovative therapy that has the potential to alleviate clinical symptoms by changing processes of brain regulation (Luctkar-Flude and Groll, 2015). This non-invasive training allows real-time processing of EEG signals, extraction of parameters of interest, and subsequent visual or auditory feedback (Hetkamp et al., 2019; Micoulaud-Franchi et al., 2015). Behavioral changes can be achieved by modulating brain activity, e.g., through volitional control (Micoulaud-Franchi et al., 2015). NF is already widely used in the treatment of attention and hyperactivity disorders, affective disorders, strokes, epilepsy, migraine, and chronic insomnia. A recent review strongly corroborates the effectiveness of NF on affective cancer related impairments (Hetkamp et al., 2019). Another publication of our working group was able to show the effects on psychological distress and affective symptoms and quality of life of NF in comparison to mindfulness-based treatment (Fink et al., 2023). Current literature shows a large emotional burden in cancer patients, while there is evidence of the benefits of NF and mindfulness treatment in this cohort of patients.

To gain a more detailed understanding of emotion processing in cancer patients the aim of this substudy of a large RCT investigation (already published in Integrative Cancer Therapies) (Fink et al., 2023) was to characterize EPN and conceivably differentiate the influence of the two different treatment options, EEG NF and mindfulness, on EPN in an existing cancer cohort. Since these treatment options are promising, we hypothesize that the 5-week NF or mindfulness intervention influences the EPN in cancer patients.

## 2 Materials and methods

This waitlist-controlled, clinical study is part of a larger project (registration in the German Register of Clinical Studies: DRKS00015773). The study was approved by the Ethics Committee of the Medical Faculty of the University of Duisburg-Essen (No. 18-8079-BO) and all participants gave written informed consent.

### 2.1 Procedure and participants

**Figure 1:**
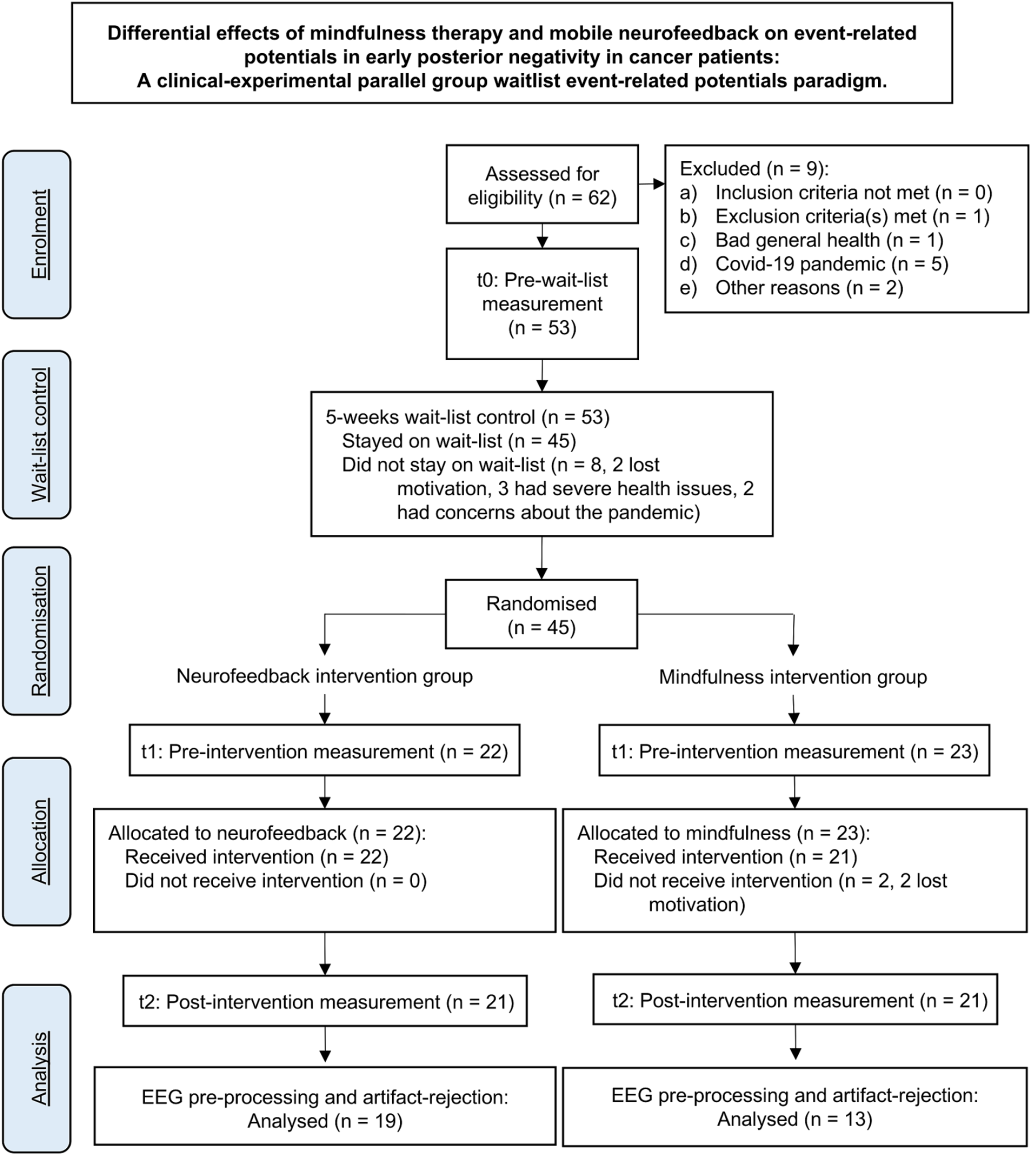
Consort flow chart.

Participants were recruited at the West German Cancer Center (Westdeutsches-Tumor-Zentrum, WTZ), the comprehensive cancer center of the University Hospital Essen, via social media, and common local newspapers. A total of 56 of initially 62 interested patients were included. Inclusion criteria were age between 18 and 70 years and the diagnosis of a malignant tumor disease. Exclusion criteria were a major depressive episode (F 32.2 or F33.2, F33.3) according to the ICD-10 checklist (Dilling and Freyberger, 2019), acute suicidality, psychotic symptoms or illness, and central nervous disorders. Since the study was conducted in German language only, participants with poor knowledge of the German language were excluded. One patient was excluded due to an existing alcohol dependence syndrome [F10.2; (Dilling and Freyberger, 2019)]. The drop-out rate was 25% partly due to the Corona pandemic, so 28 female patients (*M* = 50.07 years; SD = 9.014; range = 31–61 years) and 14 male patients (*M* = 54 years; SD = 10.379; range = 32–73 years) underwent the RCT. The according CONSORT flowchart is shown in Figure 1.

One patient reported an incorrect age at the time of inclusion; data from this patient were not excluded from the analyses in accordance with the intention-to-treat principle. The demographic data for the total cohort as well as for the two randomized, stratified subcohorts (NF and mindfulness) can be found in Table 1.

**Table 1:** Demographic data of the participants.

	Total *N* = 33		
	Waitlist	Neurofeedback	Mindfulness
	*N* = 33	*N* = 19	*N* = 14
**Sex**			
Female	23 (69.7) 76.5	13 (68.4)	10 (71.4)
Male	10 (30.3) 23.5	6 (31.6)	4 (28.6)
Age Mean (SD; range) [Years] (SD, range)	51.91 (10.004; 31–73)	52.47 (10.961; 31–73)	51.14 (8.883; 32–67)
**Relationship**			
Alone	10 (30.3) 23.	4 (21.1)	6 (42.9)
With partner	23 (69.7) 76.5	15 (78.9)	8 (57.1)
**Education**			
High school diploma	14 (42.4) 53.0	9 (47.4)	5 (35.7)
Secondary school degree (“*Realschule*“)	12 (36.4)	5 (26.4)	7 (50.0)
Secondary school degree (“*Hauptschule*“)	4 (12.1)	2 (10.5)	2 (14.3)
Missing	3 (7.1) 11,8	3 (15.8)	
**Cancer type**			
Breast	10 (30.3)	6 (31.6)	4 (28.6)
Melanoma	3 (9.1)	3 (15.8)	
Lung	3 (9.1)	2 (10.5)	1 (7.1)
Head–neck-tumor	2 (6.1)	1 (5.3)	1 (7.1)
Gastrointestinal	5 (15.2)	2 (10.5)	3 (21.4)
Hematological	7 (21.2)	3 (15.8)	4 (28.6)
Others	3 (9.1)	2 (10.5)	1 (7.1)
**Tumor stage (UICC)**			
I	4 (12.1)	3 (15.8) 1 (4,8)	1 (7.1)
II	1 (3.0)		1 (7.1)
III	12 (36.4)	8 (42.1)	4 (28.6)
IV	16 (48.5)	8 (42.1)	8 (57.1)
Median	3	3	4
Mean (SD)	3.21 (0.992)	3.11 (1.049)	3.36 (0.929)

The sample was stratified for tumor stage [*U*(41) = 220.5, *p* = 1.0]. Gastrointestinal = pancreas, rectum, intestine, gallbladder; hematological = lymphoma, multiple myeloma, leukemia; others = angiosarcoma, seminoma, ovarial.

Completers were defined *a priori* as patients who participated in six or more of 10 intervention sessions. None of the patients were non-completers. One patient dropped out of the study after the sixth NF session due to somatic deterioration, so that this patient’s data could not be included in the analyses.

Initial demographic data were collected, and a first EEG was conducted at study entry (time point T0). After a waiting period of 5 weeks, participants completed a second EEG (time point T1) and were randomized into a 5-weeks NF or mindfulness intervention. An EEG was conducted again after the intervention (time point T2). For ethical reasons, all patients received an intervention after the waiting phase, so that data gathered from T0 to T1 were considered as waitlist control phase and those gathered from T1 to T2 as intervention phase.

### 2.2 EEG acquisition

EEG was recorded using a 32-channel Brain Products Inc. (Gilching, Germany) DC EEG amplifier and matching hoods with active, sintered Ag-AgCl electrodes (Acti-Cap slim). Brain Products software (Brain Products Recorder, Version 2) was used for EEG data acquisition. EEG was recorded at a sampling rate of 1,000 Hz in the frequency range from 0.016 to 250 Hz across all 32 channels according to the extended 10–20 system: FP1, FP2, F7, F3, Fz, F4, F8, FC7, FC3, FCz, FC4, FC8, T3, C3, Cz, C4, T4, TP5, CP3, CPz, CP4, TP6, T5, P3, Pz, P4, T6, Oz, and left and right earlobes. To control for eye movements, horizontal EOG was recorded from two electrodes at the left and right outer canthi of both eyes. Vertical EOG was recorded from below the left eye and electrode Fp2. Electrode Fz served as reference, the ground electrode was mounted between Fz and FPz. Visual stimuli and patients’ responses were managed using Presentation software (Version 20.2, Neurobehavioral Systems, Inc., CA, USA). Stimulus events and responses were registered by the EEG recording software.

### 2.3 Oddball task

Patients were presented with a picture set consisting of pictures from the International Affective Picture System (IAPS; Lang and Bradley, 2007). The content of the target stimuli differed in valence (positive vs. negative) and arousal (high vs. low), forming four categories: low-positive, high-positive, low-negative, high-negative. We applied 560 standard stimuli (75%), and 35 deviants for each emotion condition (6.25%). Deviants being positive and negative valence and high and low arousal. This adds to a total of 700 stimuli. With 2.5 s SOA the presentation lasted about 30 min. We apology for imprecision in our previous description, which was due to the development of the stimulation in the planning and testing phase of our study. In case of summation of artifacts or technical problems we added additional runs of the presentation procedure to achieve more robust data, on other cases subjects presented signs of distress and the trial presentation had to be shortened for them. We added the number of trials per subject to the methods section. According to the instruction. All neutral pictures were non-target trials.

Patients were instructed to press one of two different mouse keys (randomized for left and right) to differentiate between positive and negative emotional images, while no reaction was required to neutral stimuli.

IAPS provides valence and arousal ratings on a scale from 1 (very unpleasant and very relaxing, respectively) to 9 (very pleasant and very exciting, respectively, see Supplementary material for numbers of used pictures). Mean (*SD*) arousal and valence ratings, respectively, were as follows: neutral = 3.034 (0.561) and 5.124 (0.349), low-arousal-negative-valence = 4.512 (0.345) and 2.86 (0.31), high-arousal-negative-valence = 6.483 (0.321) and 2.87 (0.45); low-arousal-positive-valence = 4.44 (0.251) and 7.232 (0.223); high-arousal-positive-valence = 6.547 (0.399) and 7.158 (0.372). Current picture sets significantly differed in valence, *F*(4,12) = 13454.8, *p* ≤ 0.05, as well as high- and low-arousal picture sets both significantly differed from the neutral pictures, *F*(4,3) = 41242.9, *p* ≤ 0.05.

### 2.4 EEG procedure

The EEG was conducted in a dimly lighted room, minimized for electrical artifacts. Patients sat on a comfortable chair 1.5 meters from a monitor, on which the stimuli were presented. EEG electrodes were attached to the patient’s heads. Patients were asked to avoid blinking and focus on the centered fixation cross on the screen between stimulus presentations.

### 2.5 EEG data analyses

Artifacts in the EEG data, resulting from patient’s jaw or eye muscle movements, were excluded from further analysis. After re-referencing the data to left and right earlobes as new reference, a bandpass finite impulse response filter between 0.032 and 30 Hz was applied (Makeig et al., 1997). Using independent component analysis as implemented in the Analyzer Software eye blinks and eye-movements were corrected. EEG-data were then averaged for each individual and each stimulus condition (neutral, low- and high-arousal-positive-valence and low- and high-arousal-negative-valence stimuli) following stimulus category specific segmentation (−100 to 1,000 ms) and baseline-correction of segments (−100 to 0 ms). From the single-subject averages, mean amplitudes of the EPN were identified between 230 and 290 ms and exported for further statistical analyses. EPN Data of 32 patients (*N*_NF_ = 19, *N*_Mindfulness_ = 14) were included in the analysis.

### 2.6 Neurofeedback treatment

**Figure 2:**
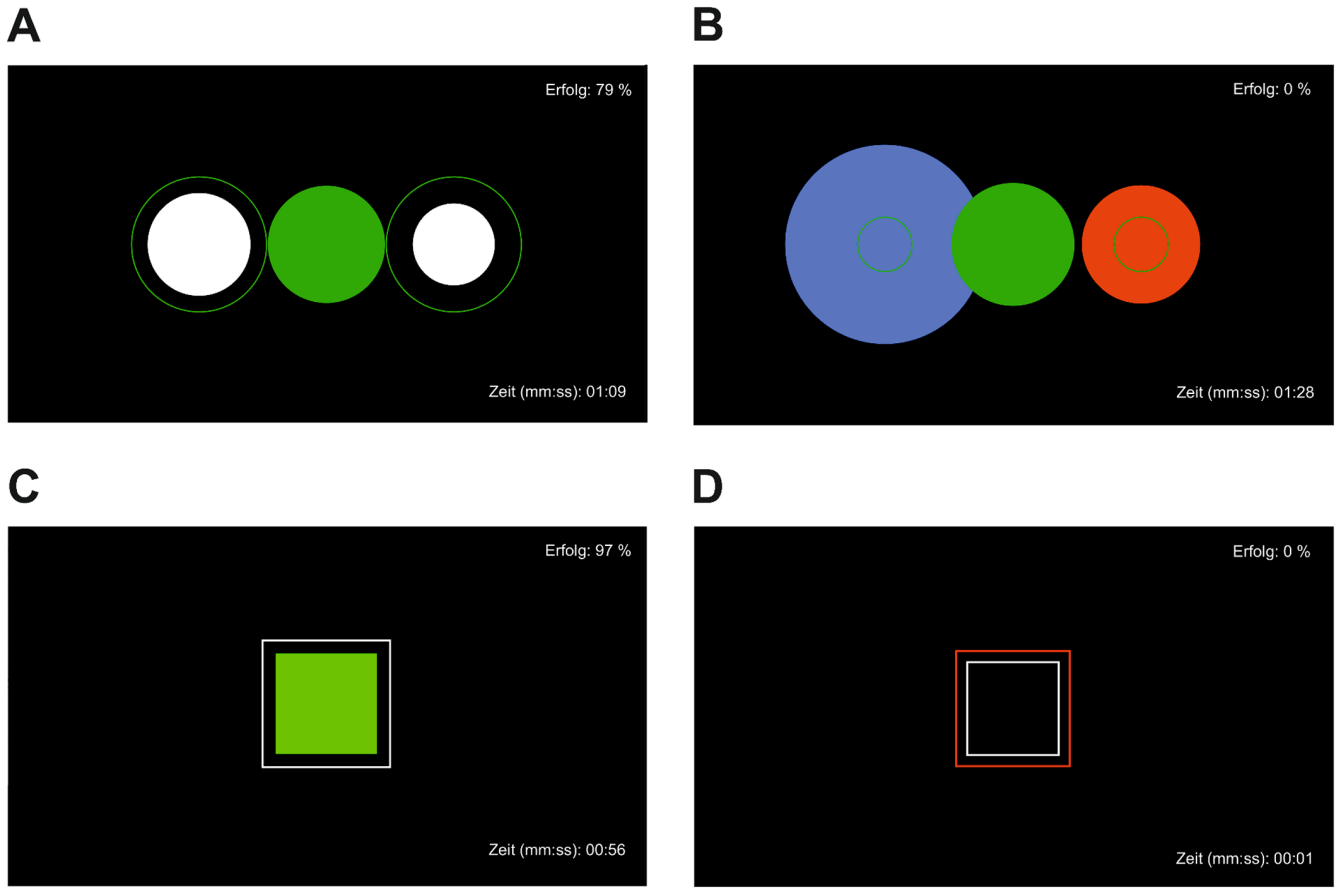
Visual neurofeedback with success rate displayed in the upper left corner and time since the beginning of the exercise in the lower left corner. **(A)** Achieving target state relaxation, **(B)** target state relaxation not achieved, **(C)** achieving target state attention, **(D)** target state attention not achieved.

The mobile NF treatment was performed using a modified *Mind Wave* headset (NeuroSky & Inc., 2011) and positioning the active electrode at coordinate Cz, reference and ground electrodes on the ear clip. For visualization the *BioEra Pro* software was used to transmit and process the recorded signal from the headset to the computer. Using *Fast Fourier Transformation based filter algorithms*, the raw signals were decomposed into individual frequency bands. This output signal was used as feedback displayed on the monitor as the target condition during training (Figure 2). As stimuli, subjects saw geometric figures, which changed depending on the degree of match with the target condition. Stimuli were either circles or squares changing their colors from green and white to blue and red indicating high or low accordance, to the target condition, respectively. The success rate was displayed in the upper right corner and elapsed time during the exercise in the lower right corner of the screen. The investigator (MF) was present throughout the training but did not provide verbal feedback or performed any manipulation of the feedback process. The NF intervention included a minimum of six and a maximum of ten training sessions, each lasting 40–45 min. The sessions took place in the outpatient clinic, twice a week over a period of 5 weeks and followed the following structure: resting state for approximately 5 min, alpha training (9–13 Hz attenuation) and reduction of theta/beta (>20 Hz) for 10 min, resting state for approximately 5 min, target theta/beta ratio ≤ 2.5 for approximately 5 min, resting state for approximately 5 min, alpha training (9–13 Hz attenuation) and reduction of theta/beta (>20 Hz) for 10 min. The conducted NF training was chosen due to several reasons. While central frontal theta and beta activity are associated with arousal (Strijkstra et al., 2003; Haenschel et al., 2000), occipital activity is proposed as a surrogate marker for a relaxed state (Niedermeyer, 1997). The objective of theta/beta NF training is to decrease theta/beta frequency to reduce arousal, while alpha NF training aims to increase alpha frequency to induce a relaxed brain state. Consequently, alpha and theta/beta NF techniques are frequently employed and have shown effectiveness in treating symptoms such as anxiety, depression, and fatigue, which are also common among cancer patients (Hetkamp et al., 2019).

### 2.7 Mindfulness treatment

The investigator (MF), who is trained in mindfulness and relaxation exercises, conducted the mindfulness intervention, analogously to the NF intervention twice a week. Based on various evidence-based German mindfulness programs (Lohmann and Annies, 2018; Teasdale et al., 2015), different exercises were included in the manual for this study. This intervention was conducted as a pre-manualized set of suggested exercises (with Mindful Sitting Meditation as basic exercise) to achieve a training duration of 35–40 min per session, analogous to the NF sessions. The groups ranged in size from two to six patients.

### 2.8 Statistical analysis

Statistical analysis was performed using the Statistical Program for Social Sciences SPSS version 26 (IBM, New York). Figures were created using Prism 9.0.2 (GraphPad, San Diego). For all analyses, the significance level was set at *α* = 0.05. All analyses were conducted after outlier-correction (± one standard deviation (*SD*)) using graphical analysis via boxplots. The electrodes Oz, O1, and O2 were added up to form a new occipital sensor (OS) as the basis for the following analyses. To test the hypotheses, mixed analyses of variance (mixed ANOVA) were conducted. The three measurement time points (T0, T1, and T2), as well as valence (positive vs. negative) and arousal (high vs. low) were included as within-subject variables. Intervention (NF vs. mindfulness) was added as a between-subject variable. If analyses were significant, a second mixed ANOVA, including only T1 and T2, was conducted to test further intervention effects. If sphericity was violated, we would report Greenhouse–Geisser corrected values. Group comparisons between treatments ΔT2-T1 and waitlist (WL-CG, NF and mindfulness) were performed using Wilcoxon tests and Monte-Carlo correction. Post-hoc tests were Bonferroni-corrected. For each stimulus condition, mean response times over all stimulus trials were computed. Response times (from the time the stimulus appears to the button-press response), that fell within 2.5 *SD* were included in the analyses. To test the effects of valence and arousal an ANOVA with the factors valence (positive, negative) and arousal (low, high) on the mean reaction time was conducted. The *SD* is given in parentheses below.

## 3 Results

### 3.1 Participants

There were no group differences for sex [*U*(41) = −0.647; *p* = 0.518] or age [*U*(41) = 176.0; *p* = 0.262]. The sample was stratified for tumor stage [*U*(41) = 220.5, *p* = 1.0]. All patients had the diagnosis of an adjustment disorder as a maladaptive response to the cancer disease.

### 3.2 Behavioral data

Mean stimulus response times for positive, negative, high arousal, and low arousal stimuli during the oddball task at the three measurement time points (pre-waitlist, pre and post intervention) are reported in Table 2. The repeated measures ANOVA did not reveal significant differences between the three measurement time points [*F*(1.668,48.378) = 1.88, *p* = 0.169].

**Table 2:** Mean response times (ms) across stimulus categories and measurement time points.

	Pre-waitlist	Pre-intervention	Post-intervention
	*N* = 33 (%)	*N* = 33 (%)	*N* = 32 (%)
Negative-low (ms)	622.588 (211.097)	609.229 (234.328)	501.959 (202.747)
Positive-low (ms)	626.517 (213.913)	609.279 (233.144)	501.258 (202.479)
Negative-high (ms)	973.154 (1117.376)^x^	1098.922 (2005.528)^x^	871.396 (939.044)^x^
Positive-high (ms)	624.854 (211.196)	609.977 (233.379)	497.140 (201.608)

SD is given in parentheses, ^x^ statistical outliers ≥ 2 SD.

### 3.3 EPN data

EEG data analysis was based on single subject averages of mean amplitude EPN data calculated from electrodes occipital sensor (OS) in 19 patients receiving NF training and 14 patients receiving mindfulness group treatment. We computed a repeated measures MANOVA on EPN amplitudes including the within factors time (pre-WL/pre/post), valence (positive/negative), arousal (low/high) and the between factor treatment group (mindfulness/neurofeedback). Here, we report Greenhouse–Geisser corrected results when no sphericity could be assumed. Mean amplitudes (μV) across groups and conditions are given in Table 3.

**Table 3:** Mean amplitudes (μV) across groups and conditions.

	Waitlist	Neurofeedback		Mindfulness	
	*N* = 33	*N* = 19		*N* = 14	
	Pre-WL	Pre	Post	Pre	Post
Negative-low	0.408 (0.273)	0.642 (0.590)	−0.007 (0.599)	0.256 (0.602)	0.003 (0.591)
Positive-low	−0.122 (0.339)	−1.199 (0.365)	−1.288 (0.451)	−0.945 (0.609)	−1.682 (0.423)
Negative-high	−0.393 (0.333)	−0.146 (0.575)	−0.906 (0.616)	−3.05 (2.255)	−0.688 (0.383)
Positive-high	−0.469 (0.364)	−1.414 (0.469)	−1.474 (0.442)	0.316 (1.114)	−1.569 (0.689)

The standard error is given in parentheses. All data are given in μV.

Results involving the within factor time or the between factor group revealed a main effect for time [*F*(1.860, 55.807) = 9.369, *p* < 0.001] but no general main effect of group [*F*(1, 30) = 0.071, *p* = 0.792].

**Figure 3:**
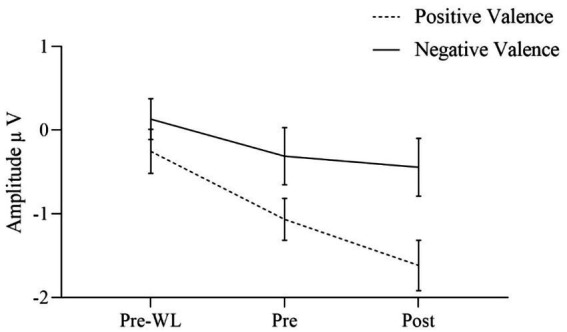
EPN for positive and negative valence target stimuli before waitlist (Pre-WL), before (Pre) and after (Post) intervention. Error bars show the 95% confidence intervals. Positive stimuli evoked more negative amplitudes than negative.

**Figure 4:**
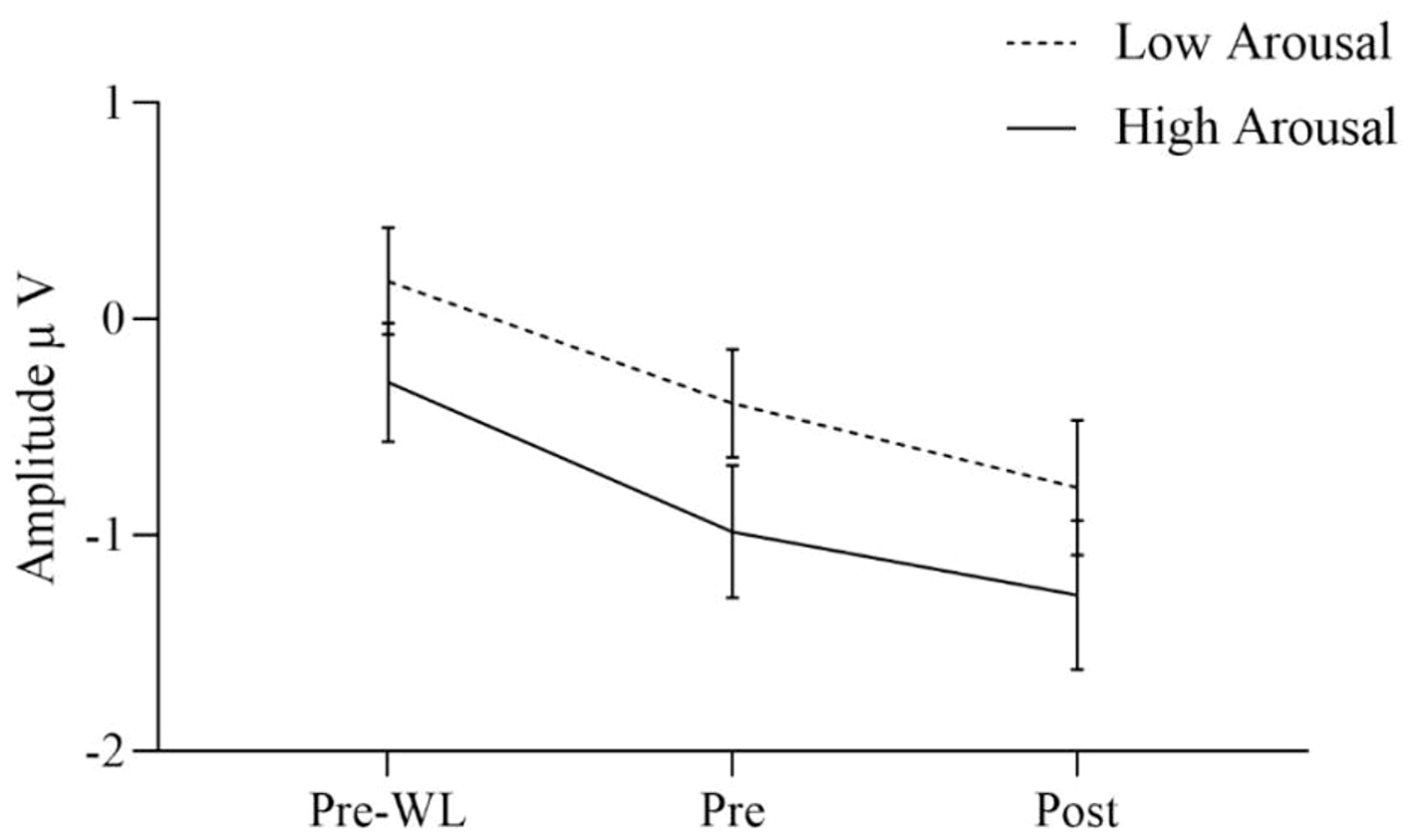
EPN for low and high arousal target stimuli before waitlist (Pre-WL), before (Pre), and after (Post) intervention. Error bars show the 95% confidence intervals. EPN after high arousal stimuli were more negative than after low arousal stimuli.

The effects of the condition factors valence and arousal are demonstrated in significant main effects for both factors. Our results indicate that the effect of valence was quite larger [*F*(1, 30) = 17.640, *p* < 0.001] as compared to the arousal effect [*F*(1, 30) = 6.753, *p* = 0.014]. Positive stimuli evoked more negative amplitudes than negative (Figure 3). EPN after high arousal stimuli were more negative than after low arousal stimuli (Figure 4).

Regarding two-way interactions we found no significant effects involving time or group [time * group: *F*(1.860, 55.807) = 0.024, *p* = 0.970, group * valence: *F*(1, 30) = 0.027, *p* = 0.871, group * arousal: *F*(1, 30) = 0.593, *p* = 0.447, time * valence: *F*(1.773, 53.204) = 1.594, *p* = 0.214, time * arousal: *F*(2, 60) = 0.052, *p* = 0.949]. However, the two-way interaction of valence * arousal was significant [*F*(1, 30) = 12.753, *p* = 0.001] with similar EPN after positive stimuli regardless of the arousal, while negative stimuli with low arousal evoked less negative amplitudes than those with high arousal. Figure 3 shows the three-way interaction of time, intervention group and valence condition [*F*(1.773, 53.204) = 2.901, *p* = 0.070]. EPN increased for both positive and negative valence target stimuli over the three measurement time points (Figure 3).

For the factor time with three measurement points we assessed polynomial decomposition of time related effects and analyzed linear and quadratic components. This revealed that the main effect of time was based on the linear [*F*(1, 30) = 23.868, *p* < 0.001] but not the quadratic component [*F*(1, 30) = 0.432, *p* = 0.516], indicating a linear overall increase of EPN amplitudes across time (i.e., more negative amplitudes). The same applied to the time * valence two-way interaction, which was not significant in the main analysis. Polynomial decomposition revealed a significant linear [*F*(1, 30) = 4.956, *p* = 0.034], but no quadratic effect [*F*(1, 30) = 0.003, *p* = 0.955] on valence amplitudes over time. The increase of EPN over time evoked by positive stimuli was larger than by negative stimuli (Figure 2). The polynomial decomposition of the three-way interaction effect of time group valence revealed quadratic [*F*(1, 30) = 4.202, *p* = 0.049] but no linear effects [*F*(1, 30 = 0.154, *p* = 0.697] within groups and valence over time. Figure 4 demonstrates that EPN amplitudes develop differently over time with regard to the processing of positive and negative emotion valence in patients treated with mindfulness or NF interventions.

**Figure 5:**
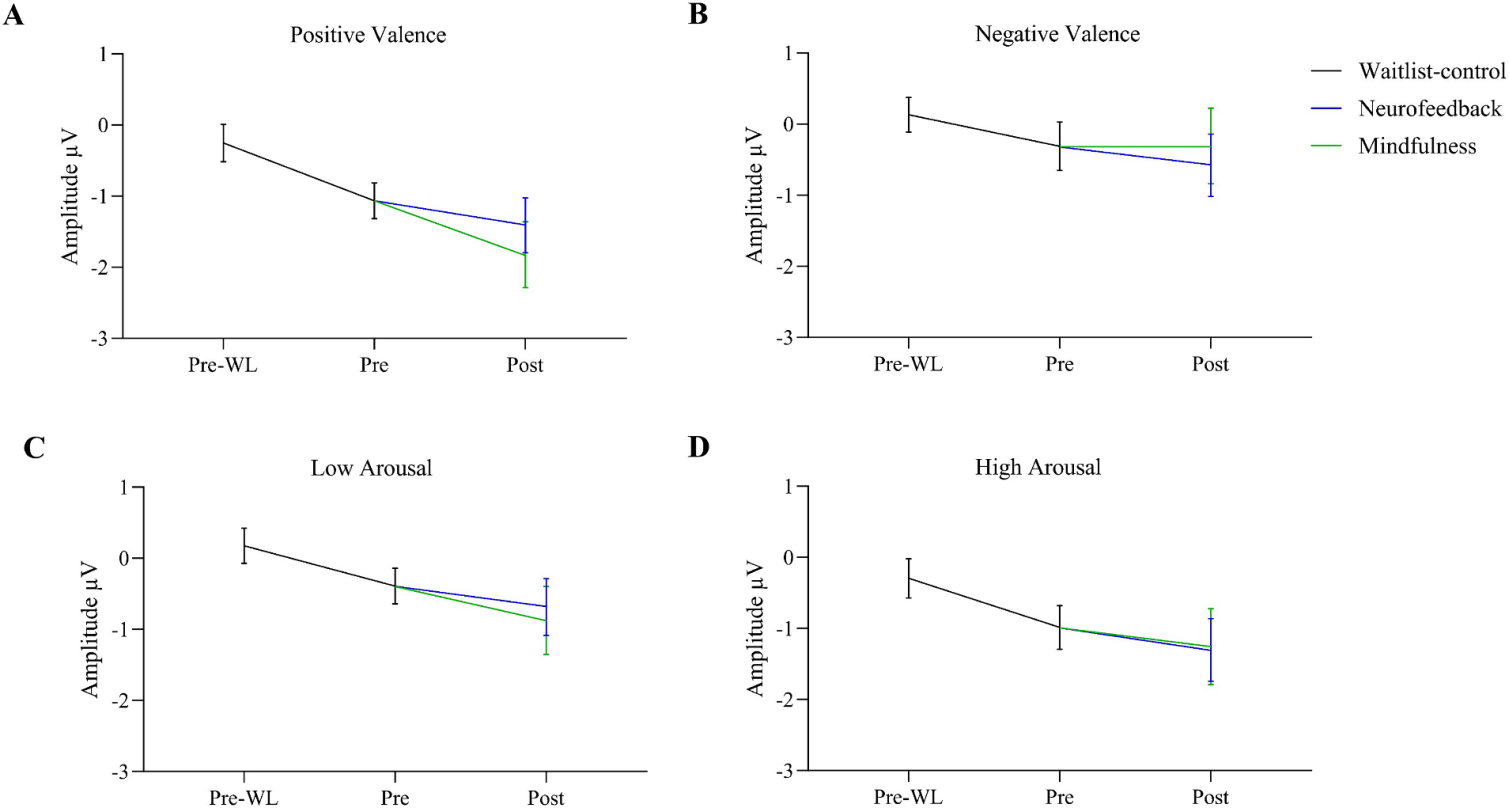
Changes in EPN after positive **(A)** and negative valence **(B)**, low **(C)** and high arousal **(D)** target stimuli during the waitlist from pre-waitlist (Pre-WL) to before the intervention (Pre) and separated for Neurofeedback and Mindfulness during the intervention from pre to post. For positive valence stimuli **(A)**, EPN increased stronger from pre to post in the mindfulness group than in the NF. For negative valence, there was a stronger increase from pre to post for NF than for the mindfulness group. Error bars show 95% confidence intervals. Grand averages regarding EPN in both groups across each condition for the three measurement time points can be found in the Supplementary Figures S1, S2.

For positive valence stimuli, EPN increased stronger from pre to post in the mindfulness group than in the NF (Figure 5A). However, for negative valence stimuli data showed a stronger increase in EPN from pre to post for NF than for the mindfulness group (Figure 5B).

## 4 Discussion

This study aimed to investigate the effects of two different psycho-oncological interventions, a NF and a mindfulness-based treatment, on emotion regulation by analyzing the specific event-related potential EPN using an oddball paradigm in cancer patients. To the best of our knowledge, this is one of the first investigations in this field. The EPN is suggested to play an important role in visual processing and shows stronger amplitudes after emotionally relevant images. Thus, changes in emotion regulation can be measured by the height (in negativity) of the EPN. Since NF and mindfulness are promising treatment options to alleviate emotional burden in cancer patients, it was hypothesized that a 5-week NF or mindfulness intervention influences the amplitudes of the EPN in cancer patients. Additionally, the study included a 5-weeks waitlist before both interventions. EPN was assessed at three different time points: before waitlist (T0) as well as pre (T1) and post intervention (T2). Visual stimuli differed in arousal (high vs. low) and valence (positive vs. negative). Only patients without central nervous disorders were included in this study.

Higher amplitudes were found for positive stimuli than for negative stimuli, regardless of intervention. Hence, this is partially consistent with the literature on EPN (Schupp and Kirmse, 2021). Our results further strenghten the idea of the EPN as a tool to monitor the processing of positive valence stimuli.

Data revealed that the EPN increased over the three measurement points both after positive and negative valence stimuli as well as after high and low emotional arousal stimuli. Regarding valence, the increase in EPN during the intervention was significantly stronger for positive than for negative stimuli. At post-intervention measurement, patients receiving the NF intervention showed a significantly higher EPN amplitude after negative valence stimuli than patients receiving mindfulness intervention. Vice versa, patients receiving mindfulness intervention showed a significantly higher EPN after positive valence stimuli in comparison to patients receiving NF. Regarding arousal, EPN increased more after high arousal than after low arousal stimuli, although not significantly. The EPN amplitude was higher after negative valence stimuli with high arousal than after negative valence stimuli with low arousal. Descriptively, compared to before the waitlist, patients showed a stronger emotional reaction to affective stimuli after the waitlist. However, no differences between WL and intervention groups after positive and negative valence stimuli were identified. Moreover, the EPN after low arousal stimuli significantly differed between WL and NF, but not between WL and mindfulness condition. No differences in EPN amplitude after high arousal stimuli were found between WL and interventions groups. Response times differed between the measurement time points with shorter reaction times after the intervention than before the waitlist, which might represent a typical effect based on habituation.

Since this is the one of the first studies investigating the effects of an intervention on emotion regulation in cancer patients, it is difficult to make clear statements regarding the change in the EPN amplitude after a NF or mindfulness intervention and to draw conclusions about affective reactions in cancer patients. Though emotion regulation tends to play a big part in patient’s adaptation and well-being (Brandão et al., 2016), there are large inconsistencies among studies about the direction of the effect of emotion regulation on psychological distress. However, the operationalization of emotion regulation differed across the studies and none of them measured changes in EPN.

The data of the present study shows an increase in EPN amplitudes over time, suggesting a change in emotion regulation. A very striking result is that patients who received NF showed a stronger EPN signal after negative valence stimuli whereas patients who received mindfulness showed stronger amplitudes after positive valence stimuli. This suggests that patients receiving NF show a greater emotional response to negative stimuli than patients receiving mindfulness, which, in turn, show a greater emotional response to positive stimuli indicating a different effect of the two interventions on emotion regulation. Cancer patients show a high prevalence to emotional burdens due to their disease accompanied with affective symptoms, such as depressive or anxiety symptoms (Deimling et al., 2006; Fink et al., 2023; Mueller et al., 2022). The EPN is a common paradigm to investigate differences in emotion regulation in clinical samples with symptoms of depression and anxiety. Research on emotion processing already revealed differences in EPN amplitudes in patient cohorts suffering from psychological disorders in comparison to controls. Thus, the EPN on schematic faces with happy and angry expressions was significantly delayed and reduced in patients with major depressive disorder compared to controls (Xin et al., 2021). Furthermore, the control group showed a significantly stronger EPN for happy than for angry faces, which did not occur in patients with a major depression. In a study focussing on ERPs in a facial recognition task and viewing of passive pictures, participants with symptoms of a burnout syndrome (emotional exhaustion and depersonalization/cynicism) showed a significantly weaker EPN in processing emotional scenes than the control group (Golonka et al., 2017). These results suggest a weaker EPN amplitude for patients with affective symptoms indicating a weaker emotional response. This can be supported by the present study, where both mindfulness and NF intervention alleviate affective symptoms such as emotional distress, anxiety, and depression (Fink et al., 2023; Schmidt et al., 2023), which might have resulted in a stronger emotional response and stronger EPN amplitudes. However, research shows that healthy controls emotionally respond stronger to positive stimuli than to negative in comparison to patients with affective symptoms (Xin et al., 2021). In a more extended sense, it could be interpreted that the mindfulness intervention is more likely to support the processing of positive information than the neurofeedback intervention used in this study, whereas NF seems to entail a different processing of images with negative valence and is therefore to be seen more in the sense of a confrontational approach. However, these results were not significant and thus need more investigation in future studies.

Even though the underlying mechanisms of the association between emotion regulation and affective symptoms have not been investigated in detail, results of a meta-analysis on cancer survivors revealed moderate positive associations between a suppressive emotion regulation and psychological distress (Baziliansky and Cohen, 2021). This suggests that patients suffering from emotional burden tend to suppress their emotions and thus, show weaker EPN amplitudes, which could function as a coping mechanism. Therefore, the interventions in the present study either helped to find a different way of dealing with emotional stimuli or represent another coping mechanism that might replace the function of emotion suppression. Still, some studies found a negative correlation, suggesting that cancer might be less threatening for people suppressing their negative emotions (Ando et al., 2011; Cohen, 2013).

Descriptively, EPN after high arousal stimuli increased more during intervention than EPN after low arousal stimuli. A recent study investigated the emotional modulation of ERPs in different behavior systems and showed larger EPN amplitudes in high-arousing and threatening areas (Schupp and Kirmse, 2021). This supports the assumption that psycho-evolutionary relevant stimuli, i.e., high-arousal stimuli, lead to stronger neuronal reactions.

### 4.1 Study limitations

This is one of the first studies investigating the effects of a NF and mindfulness intervention on emotion regulation in cancer patients. Therefore, the results have to be interpreted with caution due to a lack of existing research. However, a few limitations have to be mentioned. First, the number of subjects in the two intervention groups was not equal, with 19 patients participating in the NF group and only 14 patients participating in the mindfulness group. This complicates the interpretation of the group effects. Furthermore, no significant differences were found between the WL and the interventions, which might suggest that other factors were causing changes during intervention.

### 4.2 Clinical implications

Due to the novelty of this study context and the methodological basic character, it is difficult to make statements about the clinical implications. Future studies should further investigate emotion regulation in cancer patients compared to those suffering from depression. This could be done, for example, through randomized controlled trials. However, future research in this area could identify suitable therapeutic methods for emotion regulation. Moreover, future research in this area might indicate appropriate therapeutic procedures for emotion regulation.

## 5 Conclusion

This is the one of the first studies to examine the effect of NF and mindfulness on emotion regulation mediated by the EPN signal. The results show an influence of both mindfulness and NF treatment on emotion responses. However, the results also indicate differential effects of both interventions regarding the emotional reaction to positive and negative stimuli. The results provide preliminary insights into possible effects on emotion regulation but should be investigated in further studies.

## Data Availability

The raw data supporting the conclusions of this article will be made available by the authors, without undue reservation.
