# Supplementary material for: Differential effects of mindfulness treatment and mobile neurofeedback on event-related potentials in early posterior negativity in cancer patients: a clinical-experimental parallel group design
Source: Front Psychol. 2024 Oct 1;15:1395032. doi: 10.3389/fpsyg.2024.1395032 (PMC11473409; doi:10.3389/fpsyg.2024.1395032)
Supplement: Supplementary file 1 [file Data_Sheet_1.PDF]

Supplementary Material

1 IAPS

The IAPS identification numbers used in this study are the following:

Unpleasant pictures: 2730, 3030, 3400, 6230, 6250, 6260, 6300, 6313, 6350, 6370, 6510, 6530, 6550, 8485, 9250, 9600, 2811, 3150, 3195, 3212, 6360, 3500, 3550a, 6200, 6231, 6263, 6312, 6415, 6520, 6540, 6560, 6570, 6821, 9050, 9163, 9810, 9904, 9908, 9921, 9910, 2276, 2301, 2375a, 2750, 2900a, 3300, 7361, 9000, 9001, 9002, 9007, 9008, 9031, 9041, 9102, 9186, 9220, 9265, 9280, 9290, 9291, 9320, 9330, 9331, 9341, 9342, 9395, 9415, 9417, 9432, 9561, 9584, 9830, 9831, 9832, 9912, 9471, 9440, 9390, 6561.

Neutral pictures: 5740, 6150, 7000, 7001, 7002, 7003, 7004, 7006, 7009, 7010, 7012, 7018, 7020, 7021, 7025, 7026,7030, 7031, 7032, 7035, 7036, 7037, 7039, 7040, 7041, 7042, 7045, 7050, 7052, 7059, 7060, 7061, 7062, 7080, 7081, 7090, 7095, 7100, 7130, 7140, 7150, 7160, 7165, 7170, 7175, 7179, 7185, 7186, 7187, 7188, 7217, 7224, 7233, 7235, 7236, 7490, 7495, 7705, 7950, 7205, 7055, 7043, 7017, 7019, 7053, 7161, 7491, 7512, 7547, 5731.

Pleasant pictures: 4607, 4608, 4660, 4670, 4680, 5621, 5629, 8030, 8080, 8180, 8185, 8186, 8370, 8400, 8490, 8501, 4220, 4290, 4311, 4490, 4611, 4643, 4652, 4659, 4687, 4689, 4690, 4694, 4695, 4656, 5470, 7405, 7650, 8034, 8492, 8161, 8163, 8170, 8178, 8179, 1340, 1410, 1441, 1463, 1500, 1510, 1540, 5829, 1590, 1600, 1630, 1721, 1731, 1920, 1999, 2057, 2091, 2151, 2156, 2165, 2170, 2222, 2224, 2274, 2306, 2310, 2311, 2314, 2331, 2332, 2341, 2391, 2395, 2398, 2550, 2650, 2660, 4614, 4622, 5594.

2 Supplementary Figures

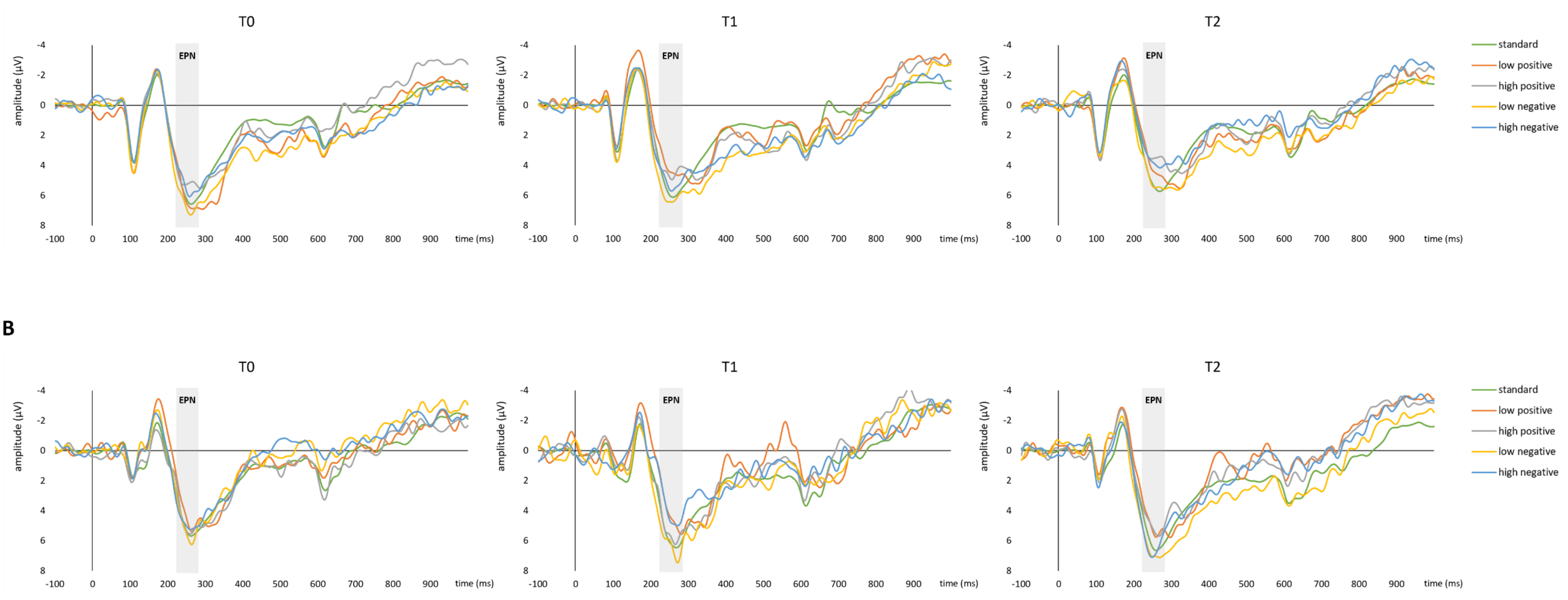

Figure 1. Grand averages of the EPN for the neurofeedback (A) and mindfulness (B) intervention across the measurement time points t0, t1 and t2 for the conditions standard, positive valence low arousal, positive valence high arousal, negative valence low arousal, and negative valence high arousal. First 100 ms display grand averages during baseline. A: N = 16; B: N = 12.

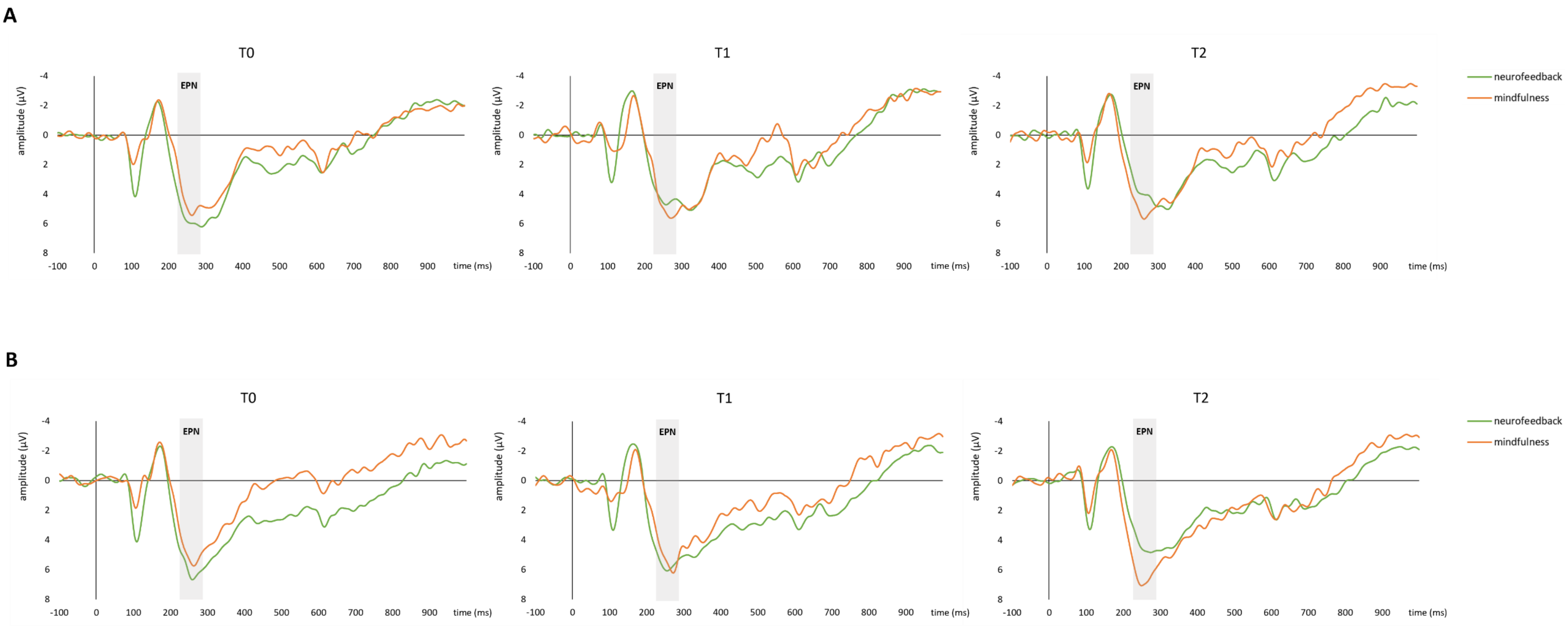

Figure 2. Grand averages of the EPN for positive (A) and negative (B) valence stimuli for both neurofeedback and mindfulness intervention across the measurement time points t0, t1 and t2. First 100 ms display grand averages during baseline.

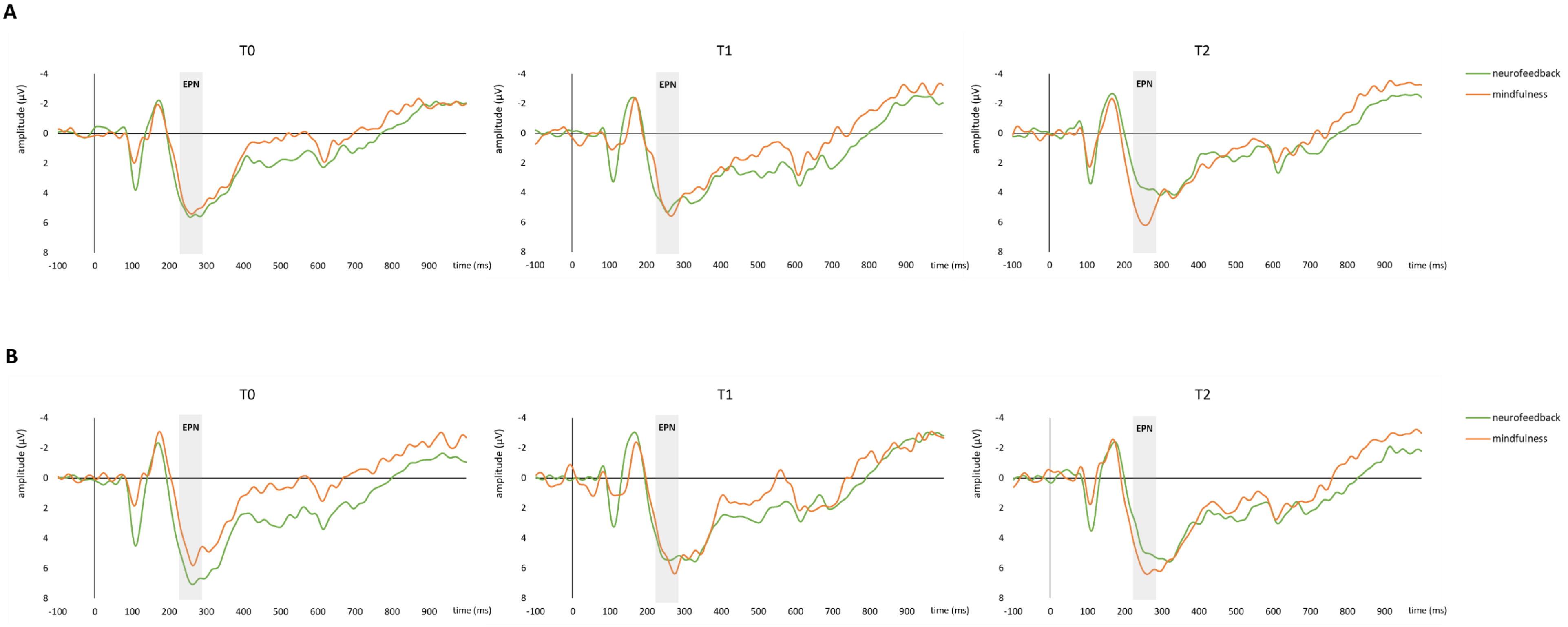

Figure 3. Grand averages of the EPN for high (A) and low (B) arousal stimuli for both neurofeedback and mindfulness intervention across the measurement time points t0, t1 and t2. First 100 ms display grand averages during baseline.
